# Recent advances in understanding the role of extracellular vesicles from probiotics in intestinal immunity signaling

**DOI:** 10.1042/BST20240150

**Published:** 2025-04-11

**Authors:** Atsushi Kurata, Koichi Uegaki

**Affiliations:** 1 Department of Applied Biological Chemistry, Faculty of Agriculture, Kindai University, Nara 631-8505, Japan; 2 Agricultural Technology and Innovation Research Institute, Kindai University, Nara 631-8505, Japan

**Keywords:** active compounds, extracellular vesicles, intestinal immunity, intestinal immune homeostasis, probiotics

## Abstract

The diverse functions of gut symbiotic bacteria are attracting attention for their potential as probiotics. Some of those bacteria release extracellular vesicles (EVs), spherical structures of approximately 20–400 nm in diameter, outside their cell bodies. Recent research has significantly advanced our understanding of the physicochemical and biochemical properties, functions, and host–cell interactions of EVs released by probiotic bacteria used in food fermentation, such as lactic acid bacteria, bifidobacteria, butyric acid bacteria, and acetic acid bacteria. However, concerns have been raised regarding the use of these EVs as postbiotics. In this review, we discuss the newly discovered roles of EVs in the gut immune signaling and the challenges associated with their application as postbiotics.

## Introduction

### Extracellular vesicles from probiotics and the gut immune system

Probiotics are defined as live microorganisms that provide health benefits on the host when administered in adequate amounts [1,2]. Extracellular membrane vesicles composed of the bacterial membrane have been identified in the culture supernatants of representative probiotics, such as lactic acid bacteria and bifidobacteria [3]. Extracellular membrane vesicles vary in size from 20 to 400 nm. Gram-negative bacteria-derived extracellular membrane vesicles are generally referred to as outer membrane vesicles, while Gram-positive bacteria-derived extracellular membrane vesicles are often called cytoplasmic membrane vesicles [4,5]. Although distinguishable nomenclature has been proposed, various extracellular vesicles (EVs) containing proteins, peptides, cell membranes, and nucleic acids from bacteria have been identified thus far. The generic term ‘extracellular vesicle (EV)’ is employed herein to refer to extracellular membrane vesicles derived from probiotics. In these bacteria, EVs are released through thinning of the cell wall and outward protrusions of the cell membrane [6]. The zeta potential of fractions containing these EVs ranges from −30 to −75 mV, indicating that EVs carry a weak negative charge [7–9]. EVs appear to readily aggregate. Several methods for the preparation of EVs from bacterial culture supernatants are mentioned in the guidelines for minimal information for studies of extracellular vesicles [10,11]. Currently, several studies have investigated the biological functions of bacterial EVs using EV-enriched fractions obtained by ultracentrifugation and density gradient centrifugation (Table 1). Filtration and chromatography are considered to be gentler alternatives [34,35]. The method for harvesting EVs is selected based on the bacterial species, downstream evaluation criteria, and research objectives. EV fractions originating from probiotics have been discovered to possess several characteristic immune-activating properties. Since the functionality of the EV fractions is involved in the mechanisms of probiotics that confer health benefits on the host, the development of technologies applying the EV fraction is anticipated.

**Table 1:** Properties of probiotic EV fractions.

Strains	Preparation of EV fractions	Experimental designs	Purported biological activities of EV fractions	References
Animal studies aimed at maintaining intestinal immune homeostasis				
*A*. *muciniphila* DSM22959	Filtration and ultracentrifugation	Oral administration of the EV fraction (20 μg protein) to gut disorder model mice	Restoration of balance of gut microbiota Enhancement of IgA production Maintenance of intestinal barrier integrity Alleviation of ulcerative colitis Therapeutic efficacy against colorectal cancer	[12]
*L*. *plantarum* NBRC15891	Filtration, ultracentrifugation, and size exclusion chromatography	Oral administration of the EV fraction (40 μg of protein) to DSS-induced colitis mouse models	Anti-inflammatory effects in DSS-induced colitis	[13]
*L*. *plantarum* KCTC11401BP	Filtration, ultracentrifugation, and density gradient centrifugation	Oral administration of EV fractions (1–100 µg of protein) to atopic dermatitis model mice	Reduction in epidermal thickening and inflammatory cytokine IL-4 level	[14]
*L*. *rhamnosus* JB-1	Filtration and ultracentrifugation	Oral administration of EV fractions (5–8 mg protein/ml) to mice	Differentiation into regulatory T cells Suppressed inflammatory responses	[15]
*L*. *paracasei*	Filtration and ultracentrifugation	Oral administration of the EV fraction (5 mg of protein) to mice with gut-induced inflammation	Protection against colitis Less weight loss, longer colons, and lower disease activity index	[16]
*L*. *reuteri* BBC3	Filtration, ultracentrifugation, and density gradient centrifugation	Oral administration of the EV fraction (200 μg of protein) to broilers	Alleviation of inflammation Minimizing intestinal injury Enhancing growth performance Lowering mortality rates	[17]
*L*. *mucosae*	Filtration and ultracentrifugation	Intraperitoneal injection of the EV fraction (50 μg) into diarrheal disease model mice	Alleviation of diarrheal disease Macrophage phenotype modulation	[18]
*L*. *kefir* KCTC 3611 *L*. *kefiranofaciens* KCTC 5075 *L*. *kefirgranum* KCTC 5086	Filtration and ultracentrifugation	Oral administration of the EV fraction (3 × 10^8^ or 3 × 10^10^ particles) to mice with gut-induced inflammation	Attenuation of inflammatory bowel disease Improvement in weight loss and reduction in bloody stool Reducing leukocyte infiltration	[19]
*C*. *butyricum* MIYAIRI II 588	Filtration and ultracentrifugation	Oral administration of the EV fraction (50 μg of protein) to mice with colitis	Protection of the intestinal barrier function Improvement of the gut microbiota Alleviation of colitis	[20]
*L*. *reuteri* DSM17938	Filtration and ultracentrifugation	Addition of the EV fraction (equivalent to 10^8^ CFU of *L*. *reuteri*/ml) to the jejunum and colon excised from mice	Reduced jejunal motility and enhanced colonic motility	[21]
Cell assay for immune cell responses				
*L*. *plantarum* JCM8341	Filtration and ultracentrifugation	Addition of EV fractions (1–10 μg protein/ml) to Peyer’s patch cells and RAW264 cells	Increased production of IgA, pro-inflammatory cytokines (IL-1, IL-6, IL-12, and IFN-γ), and anti-inflammatory cytokine IL-10	[7]
*L*. *plantarum* KCCM11179P	Filtration, ultracentrifugation, and density gradient centrifugation	Addition of the EV fraction (10 µg of protein) to THP1 cells	Transition of THP1 cells from M1 to M2 Induction of anti-inflammatory cytokine IL-10 production from THP-1 cells	[22]
*L*. *plantarum* WCFS1 *B*. *longum* *C*. *butyricum*	Filtration and ultracentrifugation	Addition of EV fractions (0.01–0.1 µg of protein) to RAW264.7 and DC2.4 cells	Production of pro-inflammatory cytokines (TNF-α and IL-6)	[23]
*L*. *sakei* NBRC15893	Filtration, ultracentrifugation, and density gradient centrifugation	Addition of the EV fraction (37 μg protein/ml) to Peyer’s patch cells	Enhancement of IgA production	[24]
*L*. *reuteri* DSM17938	Filtration and ultracentrifugation	Addition of EV fractions at ratios of 500:1, 100:1, and 20:1 (EV:cell) to peripheral blood mononuclear cells	Suppression of pro-inflammatory IFN-γ and IL-17 responses induced by T cells and NK cells	[25]
*B*. *bifidum* LMG13195	Filtration, ultracentrifugation, and density gradient centrifugation	Addition of the EV fraction (0.1 µg protein/ml) to dendritic cells	Production of anti-inflammatory cytokine IL-10	[26]
*B*. *infantis* JCM1222^T^	Filtration and ultracentrifugation	Addition of EV fractions (0.5–50 μg protein/ml) to Peyer’s patch cells and RAW264 cells	Increased production of IgA and pro-inflammatory cytokine IL-6	[9]
*B*. *longum* AO44	Filtration and ultracentrifugation	Addition of serially diluted EV fractions to splenocytes and co-cultured dendritic cells and CD4^+^ T cells	Production of anti-inflammatory cytokine IL-10	[27]
*Acetobacter* sp. WSS15	Filtration and ultracentrifugation	Addition of EV fractions (9.0–90 μg protein/ml) to RAW264 cells	Enhancement of pro-inflammatory cytokine IL-6 production	[28]
*Lactobacillus* strain RD055328	Filtration and ultracentrifugation	Addition of EV fractions (0.52–5.2 μg protein/ml) to RAW264 cells	Enhancement of pro-inflammatory cytokine IL-6 production	[8]
Protective effect against viral infection				
*L*. *crispatus* BC3 *L*. *gasseri* BC12	Filtration and ultracentrifugation	Addition of EV fraction (5 × 10^5^–5 × 10^8^ particles/ml) to human cervicovaginal and tonsillar tissues and CD4^+^ T cell lines in the presence of HIV-1	Protective effects against HIV-1 infection in human tissues and T cells by the decrease in viral entry/attachment to the target cells	[29]
Induction of apoptosis in colorectal cancer cells				
*L*. *paracasei* PC-H1	Filtration and ultracentrifugation	Subcutaneous injection of the EV fraction (200 µg of protein) into colorectal cancer model mice	Suppression of tumor growth through apoptosis	[30]
*L*. *rhamnosus* GG	Filtration and ultracentrifugation	Addition of EV fractions (50–200 µg protein/ml) to human hepatoma HepG2 cells	Apoptosis in human hepatocellular carcinoma HepG2 cells	[31]
*L*. *buchneri* HBUM07105	Filtration and ultracentrifugation	Addition of EV fractions (12.5–200 μg protein/ml) to HT-29 and human gastric adenocarcinoma cell line AGS	Induction of apoptosis through increased expression of pro-apoptotic genes	[32]
*B*. *longum* KACC 91563	Filtration, ultracentrifugation, and density gradient centrifugation	Addition of the EV fraction (2 µg protein/ml) to T cells, B cells, eosinophils, and mast cells from food allergy model mice	Reduction in food allergy symptoms by inducing apoptosis of mast cells	[33]

EV, extracellular vesicle. DSS, dextran sodium sulfate. IgA, immunoglobulin A. NK, natural killer.

Animals possess a distinct immune system in their intestinal tracts. Since a diverse community of bacteria inhabits the animal intestine, animals are shielded from potential pathogens by defensive mechanisms in the mucus layer, enterocytes, and lamina propria [36,37]. M cells are specialized epithelial cells located in the follicle-associated epithelium overlying lymphoid follicles within gut-associated lymphoid tissue. M cells play a crucial role in directly sampling antigenic substances, including gut commensal bacteria from the intestinal lumen into the Peyer’s patches [38–40]. In the Peyer’s patches, the transported bacteria subsequently interact with dendritic cells, T cells, B cells, and other immune cells to trigger an immune response [41–43]. Innate immune cells, such as macrophages, utilize receptors, including toll-like receptors (TLRs) and nod-like receptors (NLRs), to recognize and respond to various bacterial components [44,45]. In this recognition process, bacterial components induce the activation of the transcription factor NF-κB, thereby promoting the production of various cytokines. These cytokines and other activating factors stimulate acquired immune cells, including B cells, to induce the production of immunoglobulin A (IgA), while also contributing to the activation of T cells. IgA is the predominant Ig produced and secreted by the intestinal immune system [46,47]. It plays a crucial role in defense against pathogens by coating bacteria and viruses, thereby reducing their motility, inhibiting their proliferation through aggregation, and promoting their immunological clearance from the gastrointestinal tract [48,49]. In contrast, IgA was shown to promote the colonization by key commensals including *Bacteroides thetaiotaomicron* and *Bacteroides fragilis* in the gastrointestinal tract [50,51]. Stimulation by bacterial components maintains immune homeostasis in the animal intestine through the antibody IgA.

Several bacterial components such as lipoproteins, lipoteichoic acid, lipopolysaccharide (LPS), and nucleic acids are known to be recognized by TLRs and NLRs as immune activators [52,53]. However, the roles and mechanisms of these bacterial components in the increased production of IgA remain largely unknown. Although IgA production is beneficial to the host, bacterial taxonomic similarity may not be a determining factor in host-mediated gut IgA production [54,55]. In this context, it has been discovered that bacterial EVs contain these bacterial components [4,7,56], and particularly, EVs derived from probiotics are transported from the intestinal lumen to Peyer’s patches, similar to the probiotic cell bodies [12,15,57]. Oral administration of probiotic EVs maintains intestinal immune homeostasis [12,13,15]. Actually, the EV fraction derived from *Lactiplantibacillus plantarum* stimulates both innate and adaptive immune responses, resulting in increased IgA production [7]. The EV fraction from *Bifidobacterium infantis* has been shown to have a similar function [9]. In each of these EV fractions, a novel lipoprotein has been identified as an active compound involved in IgA production, respectively. Consequently, bacterial EVs are increasingly recognized as the primary vehicles for transporting the bacterial components.

This review summarizes recent findings on the mechanisms and applications of the immunomodulatory effects of EVs derived from representative probiotics. In particular, we focus on the activation of immune cells, induction of IgA production, active compounds in EV fractions, and concerns related to the application of probiotic-derived EVs.

### Regulation of biological functions by EV vesicle fractions derived from probiotics

**Figure 1:**
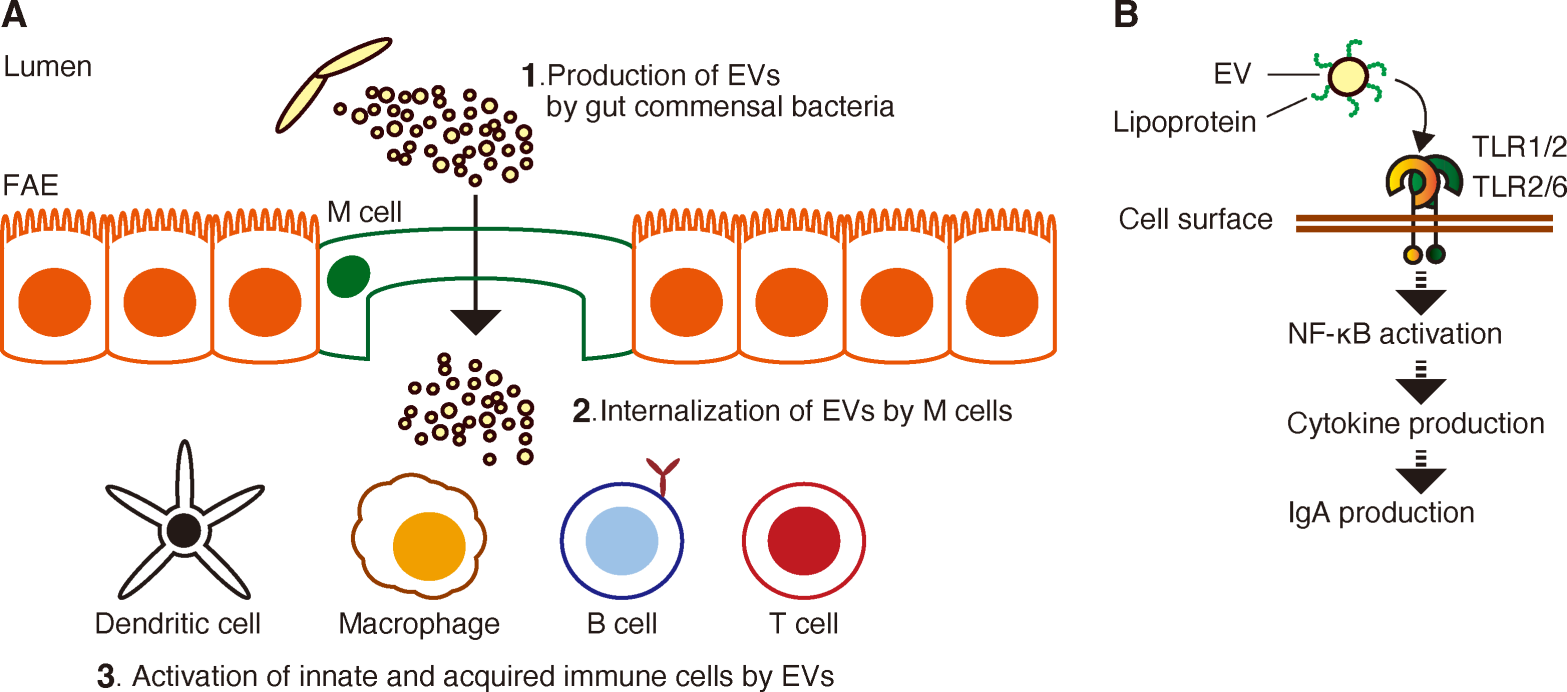
Activation of the gut immune system by probiotic EVs. (**A**) EVs released by probiotics in the intestinal lumen are taken up by M cells of FAE and activate both innate and acquired immune cells present in Peyer’s patches [15,57]. (**B**) EV lipoproteins trigger the production of pro-inflammatory and anti-inflammatory cytokines and IgA through the activation of NF-κB via recognition by TLR-2 on the surface of immune cells [7,9,23]. EV, extracellular vesicle; FAE, follicle-associated epithelium; IgA, immunoglobulin A.

It has been revealed that EVs exist in the intestinal tract, and it is becoming clear that these EVs influence a variety of biological functions. Mouse gut contents harbored 1.2 × 10^13^ nanoparticles/g with an average size of 160 nm, while nanoparticles detected in feces had an average size of 118 nm [7,58]. Certain dietary patterns may influence the EV profile derived from the gut microbiota, and dietary habits may affect the progression of metabolic diseases by forming EVs derived from the gut microbiota [59]. Mice on a high-protein diet have gut microbiota that produce significantly more EVs than those fed high-carbohydrate or high-fat diets [60]. Similar to probiotic cells, probiotic EVs (~20–400 nm in size) are taken up into Peyer’s patches from the intestinal lumen (Figure 1A). The oral administration of respective EV fractions from *Lacticaseibacillus rhamnosus* JB-1 and *Lactobacillus sakei* NBRC15893 led to EV uptake in Peyer’s patches from the mouse intestinal lumen [15,57]. It appears that these EVs are activating dendritic cells in the lamina propria. Since we consume probiotics daily, there is a growing interest in understanding how EV fractions derived from these probiotics affect our body.

Table 1 presents the results of animal and cell-based studies on an EV-enriched fraction from probiotics, evaluating its biological effects and cellular responses. A majority of studies utilize EV fractions primarily isolated by filtration or ultracentrifugation. Besides these techniques, density gradient centrifugation and size exclusion chromatography are utilized. In animal studies using mice and broilers, oral administration of probiotic EV fraction (protein content: 20 µg to 5 mg) demonstrated beneficial effects, including the maintenance of gut microbiota diversity, restoration of the intestinal barrier, and improvement of local intestinal inflammation through the regulation of both innate and adaptive immune responses. Additionally, it ameliorated systemic atopic dermatitis and enhanced growth performance.

A detailed mechanistic study of the cellular responses underlying these biological phenotypes was conducted. Using EV fraction (1–10 µg protein/ml) from *L. plantarum* JCM8341, the mechanisms underlying the activation of both adaptive and innate immunity, as well as the induction of mucosal IgA antibody production, have been revealed. The findings suggest that the induced production of pro-inflammatory cytokines, anti-inflammatory cytokines, and IgA led to the improvement of local intestinal inflammation through the maintenance of the gut microbiota diversity, restoration of the intestinal barrier, and regulation of both innate and adaptive immunities. Similar findings have been reported for other probiotic EV fractions (Table 1). The balance of local pro-inflammatory and anti-inflammatory cytokines may have regulated the systemic immune system, leading to improved atopic dermatitis [14]. EV fractions from probiotics significantly influence both local and systemic immune homeostasis by appropriately modulating the production of pro- and anti-inflammatory cytokines and antibodies. However, the recognition and neutralization of EVs and active components in bacterial EV fraction by IgA in the animal intestinal tract remain poorly understood. It is necessary to examine the effects of IgA on these EVs and active components in the EV fractions. While other EV fractions have been reported to improve gastrointestinal motility, the underlying mechanisms remain to be elucidated [15].

Additionally, each EV fraction, containing 2–200 µg of protein, induced apoptosis in both intestinal cancer cells and immune cells. Intestinal epithelial cells are replaced through apoptosis [61,62]. *Shigella* sp. bacteria and *Helicobacter pylori* have been shown to inhibit the induction of apoptosis when they infect intestinal epithelial cells, thereby increasing the survival efficiency of these bacteria in the intestinal tract [63,64]. On the other hand, probiotics may prevent infections by these pathogens through their induction of apoptosis in intestinal epithelial cells using EV fractions. Therefore, it is expected to elucidate the mechanism of apoptosis induced by probiotic EV fractions.

Collectively, these findings suggest that (1) probiotics in the gut release EVs, and (2) EV fractions act on the host’s gut immune system (Figure 1A). Probiotics may exert their beneficial effects on the host through active components in EV fractions by suppressing unnecessary inflammatory responses, preventing pathogen colonization and promoting commensal bacteria colonization on intestinal epithelial cells, enhancing gut motility to promote nutrient delivery to their habitat, and consequently improving their own survival efficiency.

### Active compounds in EV fractions from probiotics and their receptors

As mentioned above, probiotic-derived EV fractions directly affect host biological functions by comprehensively activating the intestinal immune system. Furthermore, these EV fractions indirectly influence host biology by modulating the gut microbiota. The EV fraction contains bacterial EVS and additional bacterial-derived compounds including proteins, nucleic acids, and cell wall components [4,7,56]. Therefore, it is assumed that multiple compounds, rather than a single compound, are acting as ligands to stimulate host receptors. On the host immune cell surface, TLR2 binds to bacterial lipoproteins and lipoteichoic acid, whereas TLR4 binds to LPS. Inside the cell, TLR3 detects bacterial nucleic acids, while nucleotide binding oligomerization domain 1 and 2 (NOD1 and NOD2) sense bacterial peptidoglycan [52,53]. For instance, EV fractions obtained from *L. plantarum* JCM8341, *B. infantis* JCM1222^T^, *Acetobacter* sp. WSS15, *L. plantarum* WCFS1, *B. longum*, and *Clostridium butyricum* can stimulate immune cells by activating TLR2 on their surface [7,9,23,28]. Probiotic-derived EV fractions could serve as a valuable resource for discovering novel ligands capable of targeting various host cell receptors, such as TLR2.

Several compounds derived from EV fractions of probiotics have been discovered as ligands for host cell receptors (Table 2). In EV fractions derived from *L. plantarum* JCM8341 and *B. infantis* JCM1222^T^, lipoproteins including lipoprotein 19180 and extracellular solute-binding protein were identified as potential candidates for surface-displayed proteins on these EVs [7,9]. Analogously, peptidoglycan-associated lipoprotein from *Acetobacter* sp. WSS15 was discovered as a comparable lipoprotein [28]. When EV fractions from either *Acetobacter* sp. WSS15 or *Lactobacillus* strain RD055328 were added to immune cells, the EVs were found to be localized on the cell surface [8,28]. This observation supports the hypothesis that lipoproteins presented on the EV surface can interact with and activate TLR2 (Figure 1B). Lipoproteins on EVs from *L. plantarum* JCM8341 and *B. infantis* JCM1222^T^ activate macrophages via TLR2, inducing IL-6 production. This IL-6 enhances B cell IgA production, which neutralizes pathogens and promotes commensal colonization in the gut. TLR2 recognition of lipoproteins on the surface of EVs derived from probiotics appears to initiate a cascade leading to the production of both pro- and anti-inflammatory cytokines, as well as enhancing antibody production. This suggests that these lipoproteins on probiotic EVs are a key bacterial component directly involved in maintaining homeostasis of the host’s intestinal immune system and preserving gut microbiota diversity.

**Table 2:** Properties of active compounds in probiotic EV fractions

Strains	Active compounds in EV fractions	Purported biological activities of active compounds	References
*L*. *plantarum* JCM8341	Lipoprotein19180	Enhancement of pro-inflammatory cytokine IL-6 production via TLR2 recognition for IgA production Production of pro-inflammatory cytokine IL-1, IL-12, and anti-inflammatory cytokine IL-10 via TLR2 recognition.	[7]
*B*. *infantis* JCM1222^T^	Extracellular solute-binding protein	Enhancement of pro-inflammatory cytokine IL-6 production via TLR2 recognition for IgA production	[9]
*Acetobacter* sp. WSS15	Peptidoglycan-associated lipoprotein	Enhancement of pro-inflammatory cytokine IL-6 production via TLR2 recognition	[28]
*Lactobacillus* strain RD055328	Glyceraldehyde-3-phosphate dehydrogenase	Enhancement of pro-inflammatory cytokine IL-6 production via TLR2 recognition	[8]
*L*. *paracasei*	Muramidases	Anti-apoptotic activity induced by the EGF/Akt pathway	[65,66]
*L*. *acidophilus* ATCC53544	Peptides derived from bacteriocins	Suppression of colony formation of opportunistic pathogens	[67]
*A*. *pasteurianus* NBRC3283	Lipid A moiety in lipopolysaccharide	Low TLR4 stimulation	[68,69]
*L*. *rhamnosus* JB‐1	Lipoteichoic acid	Inducing expression of anti-inflammatory cytokine IL-10	[70]
*L*. *plantarum* NBRC15891	Small RNA	Suppression of pro-inflammatory IL-8 production to prevent or ameliorate colitis	[13]

EV, extracellular vesicle. TLR, toll-like receptor.

Proteinous compounds containing lipoprotein would probably act as a ligand for host immune cells. The extracellular glyceraldehyde-3-phosphate dehydrogenase (GAPDH) found in the EV fraction of *Lactobacillus* strain RD055328 and muramidases in the EV fraction of *L. paracasei* can also function as immune cell activators [8,65]. Although it remains unclear whether *L. plantarum* JCM1149 produces EVs, GAPDH detected in its culture supernatant has been shown to activate immune cells [71]. Furthermore, GAPDH of *Lactobacillus* strain RD055328 is localized on the immune cell surface and stimulates TLR2 [8]. Therefore, it seems that at least extracellular proteins derived from probiotics act as ligands that stimulate immune cells. However, there are many unknown aspects regarding the functional domains of these proteins, their receptors on host cells, and their relationship with EVs A comparison between extracellular proteins derived from probiotics and those from other gut bacteria, including pathogens, is crucial. Further research in this area is warranted.

In contrast, among non-proteinous compounds, the lipid A moiety of LPS in EV fractions from *Acetobacter pasteurianus* NBRC3283 is recognized by TLR4 on the surface of immune cells [68,69]. Lipoteichoic acid in the EV fraction of *L. rhamnosus* JB-1 is likely recognized by TLR2, leading to IL-10 production by immune cells [70]. The RNA contained within the EVs from *L. plantarum* NBRC15891 exerts effects on immune cells [13]. *Staphylococcus aureus*-derived EVs contain nucleic acids and are taken up by immune cells, where they are recognized by intracellular TLR3 [72]. EVs carry various bacterial-derived compounds both inside and outside of them, and the EV fraction stimulates the host immune system in a multifaceted manner.

In summary, the functional diversity of EV fractions from probiotics can be attributed to the heterogeneity of their components. This heterogeneity is influenced by biosynthetic pathways of EVs and EV harvest methods. Additionally, the diverse recognition of EV fraction-derived bioactive compounds by host immune cell receptors contributes to the functional diversity. Further studies are required to identify the bioactive components and receptors of EVs. The development of purification techniques for EVs is essential. The physicochemical and biochemical properties, effects on host cells, and active compounds of EVs derived from gut commensal bacteria have been increasingly examined. Consequently, the development of technologies that utilize these probiotic EVs as regulatory substances for host biological functions is gaining significant interest.

### Applications of EV fractions from probiotics

Probiotics, including *Lactobacillus* and *Bifidobacterium*, as well as other microbial species (such as *Enterococcus*, *Acetobacter*, and *Escherichia coli*), have been extensively examined for their potential in preventive and/or therapeutic applications in various fields, including infectious diseases, cancer, depression, and obesity [73]. A major concern regarding probiotics is the presence of antibiotic resistance genes in some strains, which may be transferred to pathogens via horizontal gene transfer. Another concern is that the efficacy of probiotics is affected by a multitude of factors, including the temperature, pH, duration of fermentation, the nutrients used in fermentation, the presence of growth promoters and inhibitors during fermentation, the presence of other microbial species, and the water activity of the final product. Unless probiotics remain stable, the health benefits of probiotic products cannot be realized.

Postbiotics are defined as ‘non-viable microbial cells and their components that exert beneficial effects on host health’, encompassing heat-killed or lysed bacteria and their constituents [2,74]. In contrast with probiotics, non-viable postbiotics offer superior stability, a longer shelf life, and enhanced environmental tolerance, making them potentially more suitable for incorporation into food and pharmaceutical products [75]. Probiotic-derived EVs may be categorized as postbiotics due to their characteristics and physiological activities as discussed above [76,77]. Consequently, probiotic-derived EVs are expected to be useful in functional foods, nutraceuticals, and as therapeutic adjuvants. Probiotic-derived EVs are already present in traditional fermented products and potentially contribute to their beneficial immune-enhancing effects [28,65,68]. A large amount of enterobacterial EVs have been detected in the intestines and feces of mice and humans [7,14,58,78]. The EV fraction derived from *Akkermansia muciniphila* alleviates colitis by modulating the composition of the gut microbiota [12]. A variety of probiotic-derived EV fractions mitigate intestinal inflammation by modulating pro- and anti-inflammatory cytokine production in host immune cells. (Table 1). A technology utilizing probiotic EV fractions as adjuvants for vaccines targeting a wider range of antigens is currently under development [23,26]. There is a growing expectation for technologies that utilize EVs produced by probiotics as postbiotics.

Several issues need to be considered in the development of technology utilizing probiotic EVs. There is currently no standardized protocol to ensure the appropriate storage of probiotic-derived EVs for therapeutic applications. Furthermore, the establishment of a reliable method for bacterial EV purification is essential, and bacterial EV purification methods using column chromatography are currently under investigation [79,80]. Moreover, standardized methodologies for the quantification, molecular and physical characterization, and active compound identification of recovered bacterial EVs are very limited [81,82]. The demonstrated involvement of bacterial-derived EVs in horizontal gene transfer and antimicrobial resistance underscores the need for further investigation into the potential risks associated with the use of probiotic-derived EVs [83,84]. By resolving these challenges, we can develop a new technology using probiotic EVs that contributes to the maintenance of health in animals, including both humans and livestock.

To overcome these challenges, it is recommended to refer to the currently leading technologies utilizing animal-derived EVs, particularly exosomes. This is because similar technological challenges have been encountered in the field of the research for animal-derived EVs [85–87]. The application of probiotic-derived EVs including therapeutic one, similar to that of exosomes, is still in its developmental stages. Public awareness of international research and development trends regarding their safety, efficacy, and utilization remains inadequate. Commonly, active advances in therapeutic treatments are justified by scientific evidence. Therefore, strict regulation and monitoring by regulatory authorities in each nation will be crucial to safeguard patients from the potential risks associated with medical procedures utilizing probiotic-derived EV fractions based on the judgment of practicing physicians [88,89]. By effectively managing the promising findings of the probiotic-derived EV applications under appropriate regulations, this technology may be widely adopted and utilized in the future while maintaining high safety standards.

**Perspectives**

- Research efforts have provided important insights into the physiological activities, mechanisms of action, active compounds, and animal cell receptors associated with probiotic-derived extracellular vesicle (EV) fractions, with further knowledge being expected in the future.
- The active compounds in probiotic-derived EV fractions are proposed to stimulate the host’s intestinal immune system, enteric nervous system, and intestinal epithelial cell turnover, consequently maintaining intestinal immune homeostasis. Further investigations are expected to reveal more about active compounds and their modes of action.
- A comprehensive understanding of probiotic-derived EV fractions is crucial for the development of technologies for their utilization as postbiotics and the establishment of appropriate regulatory and oversight mechanisms for their broader applications.

## Abbreviations

EVs: extracellular vesicles
IgA: immunoglobulin A
LPS: lipopolysaccharide
TLR: toll-like receptor
